# Supplementary material for: Patterns of Intron Gain and Loss in Fungi
Source: PLoS Biol. 2004 Nov 30;2(12):e422. doi: 10.1371/journal.pbio.0020422 (PMC532390; doi:10.1371/journal.pbio.0020422)
Supplement: Table S1 — Also available at http://genes.mit.edu/NielsenEtAl/. (4.3 MB ZIP). [file pbio.0020422.st001.zip › NielsenEtAl/html/82.html]

AN4192.1.NCU00170.1.MG05319.1.FG06678.1


```
 CLUSTAL W (1.82) Multiple Sequence Alignments - Introns Inserted


Sequence 1: NCU00170.1	784 aa
Sequence 2: MG05319.1	655 aa
Sequence 3: FG06678.1	698 aa
Sequence 4: AN4192.1	634 aa
Alignment Length: 884 aa
Number Identitical Residues: 203 aa
Alignment Score (without introns) 11251


MG05319.1 	------------------------------------------------------------
NCU00170.1	-MRFF-----SKKSSRSSTSTDRVDADTRAGRYESTQPSGTSGPRSPYSHAKRSHSQSPP
FG06678.1 	MKNFFGTGKKPTPSPSPSSSTPPPSSSRKSATFEERDREHDSSEPPPYSSEPSSPAREKS
AN4192.1  	------------------------------------MVHSKSFFGFSRSHSTNQHQKQKS
          	                                         :    . :    .   . .

MG05319.1 	---------------------------------------MSNRGES--------------
NCU00170.1	PPPPPPKSLRPPTQTQQHRLSPTKPVFSSPSGTRSPSAKKTHRSDSQPRRDHSPPTPPPH
FG06678.1 	PSKSSRKSSRPTSTTEPTKSSRSSRLSRQSTDPGHSHSHSSRRPKYEPETHP--------
AN4192.1  	SSSSSKTSSKSSSPSKSHSRSSSSPSLPAFAARPRNSHRRSHDPDSHP------------
          	.. .. .:  ..: :.    : :.      :         :.  .  .            

MG05319.1 	MDIDREPPNG-TPASPKAQSPTPQPSSDETG------------------PADATGGSAR-
NCU00170.1	YNLPPTPPSGSTSRSASARDPTKHSSSSSSHQPRSSTSSRSSFGRHPVDPAAASDSRSRS
FG06678.1 	LNLPPEERKRLSALAAAMNGNSMDVDSEPVSGARSTTP-----------PNPKANAQAN-
AN4192.1  	LNLPPDELRRLSAMAAAAADPRSSMDIDSND------------------PRLTTPSEPQ-
          	 ::        :. :.   .     . .                     *   : . .. 

MG05319.1 	------------------------------------------------------------
NCU00170.1	TSFRRKKVDTDIHPLNLPPEQRKRFSDLSGFSVRNSMEFDNKENGAASAASPPPPPHKQQ
FG06678.1 	------------------------------------------------------------
AN4192.1  	------------------------------------------------------------
          	                                                            

MG05319.1 	---------------------PMN--------TDSDVPVPPPHKSPPGP---TPAEEAEG
NCU00170.1	PQDQQAQQKPTPPAHGSTFTVPMSNGADAAQTNDGEVPTPPPHRSQPTTPVQSPAEQAEG
FG06678.1 	------------------FSVPIPNGS----SHDDGAPPPPPHKSNPGSPTITPEEDAES
AN4192.1  	---------------------PNGE-------QFHQSPTPPPHRSTGNT------DEADS
          	                     *  .            * ****:*   .      ::*:.

MG05319.1 	FKAAGNKLFKDKEYLRAIGEYTKA1VNLVPNSAIYRSNRAAAFMSAGKFEEAFEDCKRSL
NCU00170.1	FKNEGNKFFKAKDYNQAIVHYTKA1IVLQPESATYLGNRAAAYMSAGKYKDALEDCTRAA
FG06678.1 	YKNAGNRFFKEKNYYKAIEQYSKA1VDLFPFSATYLGNRAAAYMSNGQFEHALDDCSRAA
AN4192.1  	FKLAGNKFFKDGNYNRAIEEFTKA1IELNPNNSIYRSNRAAANLAAHNYLDALEDAERAD
          	:*  **::**  :* :** .::** : * * .: * .***** ::  :: .*::*. *: 

MG05319.1 	ELDPDNSKTLLRLARIHTGLGKPDEALATFGRIRPPPSTKDMALAKEMLQHVEAAQSALK
NCU00170.1	ELDPNNPKILLRLARIYTSLGRPEEAIATFGRIQPPPSAKDMAPARDMLNYIQAAQKALQ
FG06678.1 	DYDPHNAKILLRLARIYTGLGRPEEALTTFSRIDPPPSAKDMVPAKEMLHHIQSARDILQ
AN4192.1  	ELDPGNNKILHRLSRTLTALGRPAEALEVLERMQPPASAADRQNAEKMLRFINQAKETLA
          	: ** * * * **:*  *.**:* **: .: *: **.*: *   *..**..:: *:. * 

MG05319.1 	SG---HASFVLRALDLAEKNLPYGALRPRKWQLMRGEALLKMADINSIGDAQNIAMSLLR
NCU00170.1	EGT--AASMVLHPLDMAERLLGIGASRPRKWVLMRGEALLRLGDINSLGEAQNIAMSLLR
FG06678.1 	QGNGSGMSMVLHALDLAERGLGHRVSKPRKWQLMRGEAYLLMGRENSLGEAQNIAMNLLR
AN4192.1  	ENR--GASMAVFCIDQARQLLGPGVKEPRAWTLLTAEAQLKMATGNSFGKAQDIAINMLR
          	..     *:.:  :* *.: *   . .** * *: .** * :.  **:*.**:**:.:**

MG05319.1 	INNQDPEALVLRGRSLYAQGENDKAIQHFRKAVSLDPDFKDAVKWLRVVQKLDRMKEEGN
NCU00170.1	SNSQDPEALVIRGRALYASGENDKAIQHFRKALSCDPDFKDAIKWLRVVQKLDRMKGEGN
FG06678.1 	NNNQDPEALVLRGRVLYGQGENDKAIQCFRMAINCDPDFRDAVKWLRIVQRLDRMKEEGN
AN4192.1  	DNNQDPDALLIRAKAYYGLGETDQALKSLKMCIGLDPDHREAIKLLRMLQKLTRTKEEGN
          	 *.***:**::*.:  *. **.*:*:: :: .:. ***.::*:* **::*:* * * ***

MG05319.1 	VEYKAGKWQAAIEKYSAALQVDPDNKGTNSKILQNRALAYNKLKQYDQAIIDCEKALSLD
NCU00170.1	DEYKAGRWQKALEKYTAALEIDPSNKGTNSKILQNRALCYTKLKQFDEAIADCERAISLD
FG06678.1 	ADFKAGRLQPAIEKYTNALEIDPSNKSMNSKLLQNRAQCKIKLKQYDDAIADCERAINLD
AN4192.1  	NAFKAKDYRKAIELYTEALSVDETNKDVNAKILQNRAQAYINLKEYDEAIKDCTEALRLD
          	  :**   : *:* *: **.:*  **. *:*:***** .  :**::*:** ** .*: **

MG05319.1 	PSYTKARKTKATALGLAEKWEDAVREWKSLAESEQGDRAILKELRHAELELKKSKRKDYY
NCU00170.1	PSYLKARKTKANALGLAERWEDCVREWKALQELEPEDRTIAQEVKRAELELKKSQRKDYY
FG06678.1 	PGYTKARKTKANALGGAERWDDAVKEWKAIQELEPEDRNIAKEIRRAELELKKAQRKDYY
AN4192.1  	PTYIKAQKMRAKAHGGAGNWQEAVSDYKAVAEANPGEKGIREDIRRAEFELKKAQRKDYY
          	* * **:* :*.* * * .*::.* ::*:: * :  :: * :::::**:****::*****

MG05319.1 	KILGITKDADDKEIKKAYRKLAVIHHPDKNPGDEAAEARFKDIGEAYETLSDPQ2KRERY
NCU00170.1	KILGIDKNADETQIKKAYRKLAIVHHPDKNPGDASAEARFKDISEAYETLSDSQ2KRARY
FG06678.1 	KIVGVEKNATDVEIKKAYRKMAVKLHPDKNPGDAQAEEKFKDLQEAYETLSDPQ2KRAAY
AN4192.1  	KILGVSKDATETDLKKAYRKLAIKYHPDKNREGEAGDEKFKEIGEAYETLIDPQ2KRAAY
          	**:*: *:* : ::******:*:  *****  .  .: :**:: ****** *.* **  *

MG05319.1 	DSGIDLEDPSDMFGGG-GHGMHGGIDPEILFSMMGGQGGFGGGE1GDDDDGRSSRAGESK
NCU00170.1	DSGDDLVDPSDMFGGG-MGGGMGGIDPEIIIQMMGGQGGHGFGG~GGFGG----------
FG06678.1 	DNGDDLMDPNDMFGGGGMGGGMGGIDPEILFSMMGQQGFGGGGG~GGFRSAGG-------
AN4192.1  	DNGDDLLDPADMFGGG--GFGMGGMG---GMGGMGGMGGMGGTH~INIDPSVLFN-----
          	*.* ** ** ******      **:.    :  **  *  *     .   .         

MG05319.1 	PYSDGGPGQSYHDSDPAQSAQWYAADDEERDDTEYGGRRDRYTAEKGRETTLAQLRKFWP
NCU00170.1	-FGGGG----------------FPGGGRSRGRGGFGGGGFHYQ-----------------
FG06678.1 	-FPGGGGG-----------AHFNFGGDPRQQRGGYPGG-FNFS-----------------
AN4192.1  	MMNGGGG--------------FASAGGNPFGGGQARGFPGGFPF----------------
          	   .**                  ...         *    :                  

MG05319.1 	WLAAVVVLLPPTLSFPLMFACGVMYIITRAANPATQNPGDWVEDDAH
NCU00170.1	-----------------------------------------------
FG06678.1 	-----------------------------------------------
AN4192.1  	-----------------------------------------------
          	
```
